# Strain Engineering for Enhanced TiN/TiO_2_ Hot Electron Photodetection

**DOI:** 10.3390/nano16160977

**Published:** 2026-08-09

**Authors:** Tingting Liu, Weijia Shao, Qingjia Zhou, Yiling Zhang, Yuhong Chen, Xinwei Chang, Ni Yao, Jie Li, Aijuan Zhang, Yanni Zhang

**Affiliations:** 1 School of Physics and Electronic Engineering, Xianyang Normal University, Xianyang 712000, China; 2 University Engineering Research Center of Advanced Functional Materials and Intelligent Sensing, School of Physical Science and Technology, Guangxi Normal University, Guilin 541004, China; wjshao@gxnu.edu.cn (W.S.);; 3 School of Science, Lanzhou University of Technology, Lanzhou 730050, China

**Keywords:** hot electron, photodetectors, strain engineering, TiN

## Abstract

Metal/semiconductor heterojunctions for hot carrier photodetection have garnered significant attention. However, enhancing the quantum efficiency remains a critical challenge. Introducing lattice strain into metallic materials offers a viable approach to enhance the performance of TiN/TiO_2_ hot electron photodetectors by effectively modulating their electronic structure. Herein, we investigate how strain influences the electronic structure of TiN and consequently affects the generation, transport, and injection processes of hot carriers using first-principles calculations. Subsequently, we evaluate the injection efficiency and responsivity of the TiN/TiO_2_ photodetector through Monte Carlo simulations. We find that compressive strain renders the energy bands more delocalized and reduces the density of states DOS, leading to diminished hot electron generation, especially in the high-energy region above the Schottky barrier. This reduction suppresses electron–electron scattering, thereby increasing the hot electron lifetime and mean free path. Consequently, the hot electron injection efficiency is enhanced, ultimately improving the responsivity of TiN/TiO_2_ photodetector by a factor of 1.3–2.4 over the incident photon energy range of 0.1–3 eV.

## 1. Introduction

The generation of energetic carriers (i.e., hot electrons and hot holes) via the non-radiative decay of surface plasmons has emerged as a versatile platform for various photonic technologies, including infrared photodetection, energy conversion, sensing, and biomedical imaging [1,2]. Conventional semiconductor-based photodetectors, such as Ga_2_O_3_ heterojunction devices that achieve efficient charge carrier separation via built-in electric fields, are often constrained by material bandgaps [3,4]. In contrast, plasmonic hot electron devices offer distinctive merits, including ultrafast temporal response, spectral tunability independent of material bandgaps, and operational robustness at ambient temperatures [5,6]. While noble metals such as gold have been extensively investigated for hot carrier photodetectors [7,8], titanium nitride (TiN) has been recognized as a promising refractory plasmonic alternative [9]. This is attributed to its pronounced plasmonic resonance across the visible-to-near-infrared (vis-NIR) spectrum, relatively low work function, and exceptional thermal and chemical stability [10,11,12,13]. Furthermore, TiN offers distinct advantages in terms of cost-effectiveness and CMOS compatibility compared to conventional noble metals [14]. For instance, TiN nanoparticle arrays have been demonstrated to effectively enhance the performance of Schottky photodetectors, and TiN/TiO_2_ structures leveraging Tamm plasmons have achieved remarkable responsivity [15,16]. Additionally, TiN-SiC devices serve as robust platforms for broadband photodetection in extreme radiation environments [17]. Nevertheless, the quantum efficiency of TiN-based hot carrier devices remains critically low, presenting a major bottleneck for practical applications.

In the scenario of hot electron attraction for photodetection, the electronic structures of metals play a deterministic role in hot electron generation, energy distribution, transport, and injection, which fundamentally dictates the ultimate quantum efficiency of devices [18,19]. It has been demonstrated that strain engineering constitutes an effective approach for dynamically tailoring the electronic band structure and optical response of metals. Strain engineering has been extensively validated in experimental studies. For instance, Carrascoso et al. employed flexible substrates to impose biaxial tensile strain on monolayer MoS_2_, confirming the efficacy of strain engineering in modulating charge transport properties [20]. Beyond mechanical stretching, stress engineering during thin-film deposition also constitutes a viable route to achieving high strain levels [21]. Theoretically, Zhong et al. reported that lattice expansion on ruthenium surfaces enhances surface reactivity via an upshift of the d-band center [22]. Similarly, Ma et al. demonstrated that compressive strain significantly suppresses interband transition probabilities in noble metals, thereby augmenting light absorption across the visible-to-near-infrared spectrum [23]. Furthermore, Liu et al. showed that compressive strain effectively modulates the electronic structure of Au, leading to a 1.5 to 3-fold enhancement in the responsivity and injection efficiency of Au/TiO_2_ photodetectors [24]. For TiN, epitaxial growth on a lattice-mismatched substrate naturally introduces strain [25,26]. This intrinsic strain offers a viable pathway to alter electronic structure, thereby optimizing hot carrier distribution characteristics, suppressing electron–electron scattering, and consequently prolonging hot carrier lifetimes and mean free paths. Despite its extensive maturity in catalysis and two-dimensional materials, the exploration of strain engineering in TiN/TiO_2_ hot electron photodetection remains conspicuously absent.

In this work, we use the first-principles calculations to investigate the modulation of the electronic structure and hot carrier dynamics in TiN under both triaxial compressive and tensile strains. Specifically, we apply tensile and compressive strains to analyze the resulting evolution of the electronic structure, hot carrier generation, hot carrier lifetimes and mean free paths. Based on these analyses, we construct a planar TiN/TiO_2_ hot electron photodetector model using Monte Carlo simulations to calculate the strain-dependent responsivity and injection efficiency across a photon energy range of 0.1–3.0 eV. Our results indicate that a −6% compressive strain reduces the population of high-energy hot electrons while simultaneously extending their lifetimes and mean free paths. This reduction in hot electron population is attributed to the broadening and lowering of the density of states (DOS) under −6% strain, which reduce electron–electron scattering, thereby prolonging the hot carrier lifetime and mean free path. Consequently, the implementation of 6% compressive strain is projected to enhance the responsivity and injection efficiency of the TiN/TiO_2_ photodetector by a factor of 1.3 to 2.4 within the specified spectral range, demonstrating that strain engineering is a viable strategy for boosting the performance of refractory TiN/TiO_2_ hot electron optoelectronic devices.

## 2. Calculation Methods

In this study, the electronic structures and hot carrier generation properties of TiN were investigated using the Vienna Ab initio Simulation Package (VASP) within the framework of density functional theory (DFT) [27]. Electron–-ion interactions were treated via the projector augmented wave (PAW) method [28]. For the exchange–-correlation potential, the generalized gradient approximation (GGA) was adopted with the Perdew–-Burke–-Ernzerhof functional revised for solids (PBEsol) [29]. A plane-wave kinetic energy cutoff of 520 eV was employed, and the Brillouin zone was sampled using a Monkhorst–-Pack k-point mesh of 20 × 20 × 20. Force convergence was achieved at a criterion of 0.01 eV/Å. For pristine face-centered cubic TiN, the optimized lattice parameter was determined to be 4.23 Å, which is consistent with the corresponding experimental value of 4.24 Å [30].

The distribution of hot carrier generation encompasses both indirect and direct transitions. Indirect transitions are governed by the joint electronic density of states and the Fermi–Dirac distribution function [31]; consequently, the energy distribution associated with these transitions can be described as follows:

(1) $$D\left(E\right)=\frac{\rho \left(E-h\nu \right)f\left(E-h\nu \right)\rho \left(E\right)[1-f(E)]}{\int \rho \left(E-h\nu \right)f\left(E-h\nu \right)\rho \left(E\right)[1-f(E)]dE}$$

Here, *E* denotes the energy of the electron after excitation, and *hv* represents the photon energy. The terms ρ(E−hν) and ρ(E) correspond to the DOS before and after excitation, respectively. f(E) is the Fermi–Dirac distribution function. The contribution from direct optical transitions is derived from the imaginary component of the dielectric function, denoted as $Im{\bar{\epsilon }}_{direct}$. This quantity is evaluated using the expression [32]:

(2) $$\lambda^{*}\cdot Im{\bar{\epsilon }}_{direct}\left(\omega \right)\cdot \lambda =\frac{4\pi^{2}e^{2}}{m_{e}^{2}\omega^{2}}\int_{BZ}\frac{dk}{{2\pi }^{3}}\sum_{n^{\prime }n}\left(f_{kn}-f_{kn^{\prime }}\right)\delta \left(\varepsilon_{kn^{\prime }}-\varepsilon_{kn}-h\nu \right){\left|\lambda \cdot {\langle P\rangle }_{n^{\prime }n}^{k}\right|}^{2}$$

Here, λ and ω denote the polarization vector and angular frequency of the photon, respectively. $\varepsilon_{kn}$ and $f_{kn}$ are the eigenvalues and electron occupancy of the state with wave vector ***k*** and band index *n*. The ${\langle P\rangle }_{n\prime n}^{k}$ signifies the matrix element of the momentum operator ***P***, which is obtained via the relation $P\equiv \frac{m_{e}}{i\hbar }[r,H]$. To calculate the matrix elements of ***r*** and ***H*** efficiently, we constructed maximally localized Wannier functions using the WANNIER90 package [33]. Furthermore, the Brillouin zone for the integration is sampled by randomly selected ***k*** points with a total number of up to 5 × 10^6^.

The electron–electron scattering (S_e-e_) and electron–phonon scattering (S_e-p_) rates governing hot carrier dynamics were computed using the JDFTx code with fully relativistic norm-conserving pseudopotentials [34]. The plane-wave cutoff and Fermi–Dirac smearing were set to 30 and 0.01 Hartree, respectively. To evaluate the electron–phonon coupling matrix elements, we employed maximally localized Wannier functions within a 3 × 3 × 3 supercell framework. The ***k***-point mesh for the Wannier function interpolation was set to 24 × 24 × 24.

The S_e-e_ and S_e-p_ rates are dictated by the imaginary components of their respective quasiparticle self-energies [35]. The imaginary part of the quasiparticle self-energy from S_e-e_ can be expressed as [32,36]:

(3) $$Im\Sigma_{kn}^{e-e}=\int_{BZ}\frac{dk\prime }{{\left(2\pi \right)}^{3}}\sum_{n\prime }\sum_{GG\prime }{\tilde{\rho }}_{k^{\prime }n^{\prime },\;\;kn}(G){{\tilde{\rho }}^{*}}_{k^{\prime }n^{\prime },kn}(G\prime )\times \frac{4\pi e^{2}}{{\left|k^{\prime }-k+G\right|}^{2}}Im\left[\epsilon_{GG^{\prime }}^{-1}\left(k\prime -k,\varepsilon_{kn}-\varepsilon_{k\prime n\prime }\right)\right]$$

Here, ${\tilde{\rho }}_{k^{\prime }n^{\prime },kn}$ is the plane-wave expansion of the product density $\sum_{\sigma }u_{k^{\prime }n^{\prime }}^{\sigma *}(r)u_{k^{\prime }n^{\prime }}^{\sigma }(r)$ of Bloch functions with reciprocal lattice vectors ***G***, and $\epsilon_{GG^{\prime }}^{-1}$ is the microscopic dielectric function in a plane-wave basis calculated within the random-phase approximation. For S_e-p_, the imaginary part of the quasiparticle self-energy is [32,36]:

(4) $$Im\Sigma_{kn}^{e-ph}=\pi \int_{BZ}\frac{\Omega dk\prime }{{\left(2\pi \right)}^{3}}\sum_{n\prime \alpha \pm }\left(n_{k\prime -k,\alpha }+\frac{1}{2}\mp \frac{1}{2})\times \delta (\varepsilon_{k\prime n\prime }-\varepsilon_{kn}\mp \hbar \omega_{k\prime -k,\alpha }\right){\left|g_{k\prime n\prime ,\;kn}^{k\prime -k,\alpha }\right|}^{2}$$

Here, q=k′−k and $n_{q,\alpha }$ are the wave vector and phonon occupation number, $g_{k^{\prime }n\prime ,\;kn}^{k\prime -k,\alpha }$ is the electron–phonon matrix element with electronic states, and Ω is the volume of the unit cell. The total scattering lifetime of hot carriers is $\tau_{kn}=\hbar /(2Im\Sigma_{kn})$, where $Im\Sigma_{kn}=Im\Sigma_{kn}^{e-e}+Im\Sigma_{kn}^{e-ph}$, the mean free path (MFP) is $\lambda_{kn}=\nu_{kn}\tau_{kn}$, $\nu_{kv}\equiv \frac{\partial \varepsilon_{kn}}{\partial k}$ is the velocity of hot carriers, and $\varepsilon_{kn}$ is the eigenvalue of the state.

Strain is evaluated by modulating the lattice parameters, which is defined by the formula:

(5) $$strain=\frac{a-a_{0}}{a_{0}}\times 100\%$$

where *a* and *a*_0_ are the strained and unstrained lattice parameters, respectively, and *a*_0_ is determined from the unstrained bulk lattice parameter [37].

## 3. Results and Discussion

### 3.1. Electronic Properties

**Figure 1:**
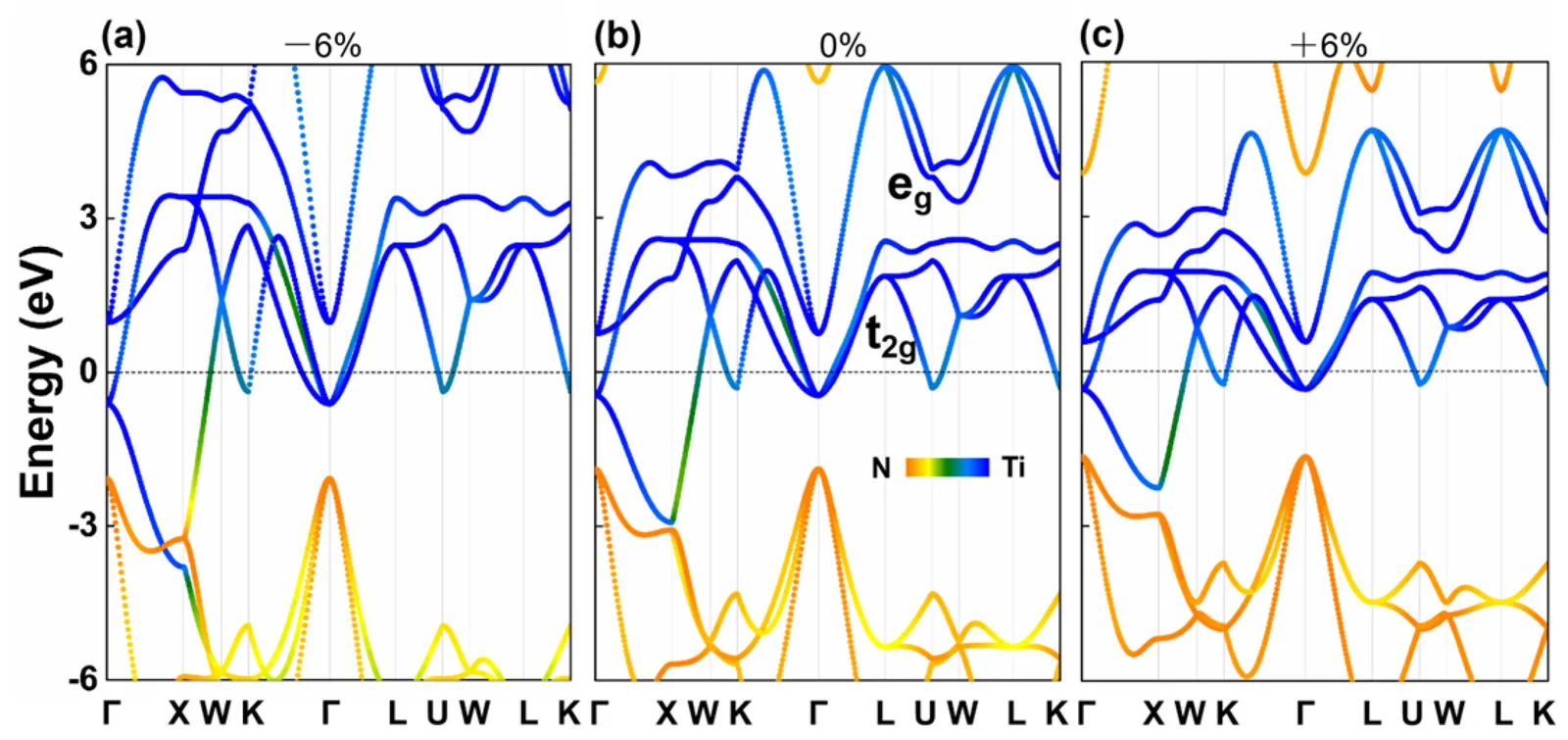
Band structures of TiN under (**a**) compressive (−6%), (**b**) pristine (0%), and (**c**) tensile (+6%) strains. The energies in (**a**–**c**) are relative to the Fermi energy level, *E*_F_.

**Figure 2:**
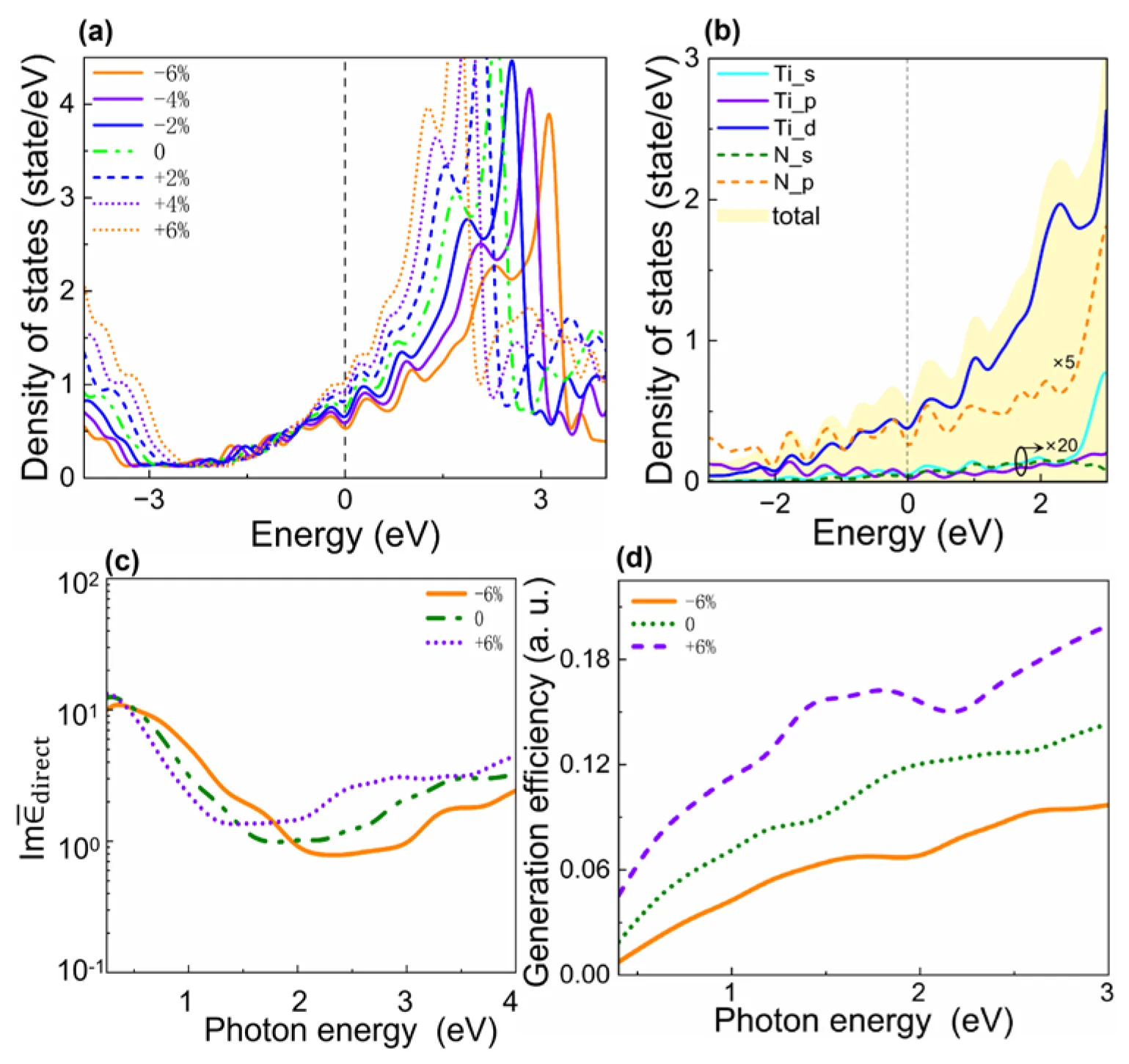
(**a**) DOSs, (**c**) imaginary part of dielectric function of the direct electric diploe transition, and (**d**) generation efficiency versus the photon energy with various strains as listed in the figures. (**b**) Projected and total (shaded region) DOSs for TiN under −6%. The DOS values for Ti_p, Ti_s, N_s and N_p bands are enlarged. The zero energy in (**a**) is set as *E*_F_.

The bulk TiN adopts the face-centered cubic (FCC) crystal structure. Each Ti atom occupies the center of an octahedron composed of its six nearest-neighbor N atoms, with each N atom residing at the analogous site—namely, the center of an octahedron formed by its six nearest-neighbor Ti atoms. Under the octahedral crystal field, the five-fold-degenerate Ti_d orbitals split into t_2g_ and e_g_ orbitals. These then hybridize separately with N_p orbitals to form bonding and antibonding pairs, which ultimately develop into energy bands under the periodic crystal potential of TiN. Considering practical feasibility, we employ a series of strains ranging from −6% (compressive) to +6% (tensile) to probe the electronic responses of TiN. Figure 1 presents the element-resolved band structures of strained and pristine TiN. Overall, the bands can be divided into two parts: the upper portion near the Fermi level is primarily contributed by Ti atoms, while the lower portion is mainly from N atoms. The bands at the Γ point essentially retain the characteristics of atomic orbitals. Allowed direct transitions occur near the Γ, W, and K points. As shown in Figure 1a, −6% strain renders the bands more delocalized. Specifically, the t_2g_ orbitals near the Γ point and the bands around the X point shift downward, whereas the e_g_ orbitals at the Γ point shift upward. This increases the energy of direct transitions, reduces the generation of hot carriers, and thereby influences the optical absorption at higher photon energies. Quantitatively, under a −6% strain, the band energy at the F point shifts downward by 0.63 eV. In contrast, Figure 2c indicates that tensile strain (+6%) results in more localized energy bands, which is beneficial for the generation of hot carriers.

Figure 2a illustrates the variation in the total density of states (DOS) with strain. Under compressive strain, the DOS decreases, whereas tensile strain increases the DOS. Additionally, under tensile strain, the DOS shifts toward lower energy regions by approximately 0.6 eV at +6% strain. For compressive strain, the DOS in the portion below the Fermi level shifts toward lower energy regions, while in the portion above the Fermi level, it shifts toward higher energy regions. This observation is consistent with the conclusion from Figure 1 that compressive strain induces increased band delocalization. Specifically, within the energy range of 0–2.5 eV, a strain of −6% (+6%) reduces (increases) the DOS by approximately 0.2–2.5 (0.2–2.7) states/eV. Furthermore, analysis of the projected density of states (PDOS) in Figure 2b reveals that the primary affected components are the Ti_d orbital and the N_p orbital. These findings indicate that the application of strain indeed exerts a significant impact on electronic transitions. This behavior originates from the compressive-strain-induced lattice shrinkage (e.g., lattice parameter from 4.23 Å to 3.98 Å; Ti–N bond length from 2.12 Å to 1.99 Å). The reduced bond length facilitates enhanced Ti_d and N_p orbital coupling, which promotes electron delocalization. This is evidenced by the steepened band dispersion, increased bandwidth, and broadened DOS. Tensile strain yields the inverse trend, characterized by diminished orbital overlap and localized electronic configurations.

### 3.2. Hot Carrier Generation

The imaginary part of the direct dielectric function ($Im{\bar{\epsilon }}_{direct}$) as a function of photon energy (*hν*) characterizes the optical absorption behavior of TiN under various strains, as shown in Figure 2c. Observations reveal that strain effects on the photon absorption of TiN are nonlinear from the visible to the near-infrared band. This nonlinearity primarily arises from electron state hybridization and modification of the momentum matrix element. Notably, under −6% strain, at photon energies 0.2–2 eV, the $Im{\bar{\epsilon }}_{direct}$ is slightly larger than that of the pristine (unstrained) case. In contrast, at photon energies 2–3 eV, the $Im{\bar{\epsilon }}_{direct}$ is slightly smaller than the pristine value. Overall, strain engineering exerts a negligible effect on the photon absorption of TiN.

Having demonstrated the electronic structure and optical properties of TiN under strain, we next focus on its strain-modulated hot carrier characteristics. Under compressive strain, the electronic states become progressively delocalized and the DOS broadens accordingly, leading to a gradual reduction in the number of carriers available for transitions. Figure 2d illustrates the relationship between hot carrier generation efficiency and photon energy under strain. The generation efficiency is defined as the ratio of the number of transition-allowed electronic states per unit energy in the Brillouin zone to the total number of electronic states. It is observed that in the near-infrared to visible spectral range, the generation efficiency under −6% strain is lower than that of the pristine (unstrained) case, especially for photon energies above 1.5 eV. This is primarily attributed to modifications in the DOS and ${\langle P\rangle }_{n\prime n}^{k}$ induced by strain effects. In contrast, the generation efficiency under +6% strain exceeds that of the pristine case, indicating that tensile strain can promote hot carrier generation. As the strain varies continuously from +6% to −6%, the generation efficiency exhibits a systematic decline across the near-infrared to visible range. This trend shift is not simply monotonic but arises from the synergistic modification of both the DOS and the ${\langle P\rangle }_{n\prime n}^{k}$. Specifically, under +6% tensile strain, the DOS near the Fermi level becomes more localized and the number of allowed transition channels increases, enhancing the efficiency. When the strain progressively shifts toward −6% compressive strain, band broadening and state delocalization gradually suppress the available transition channels. Thus, the evolution from +6% to −6% strain involves a cooperative interplay between band -structure deformation and optical selection rules, leading to a distinct suppression of hot carrier generation in the higher-energy region. However, whether a higher hot carrier yield necessarily translates into enhanced performance of hot electron detector warrants further investigation.

**Figure 3:**
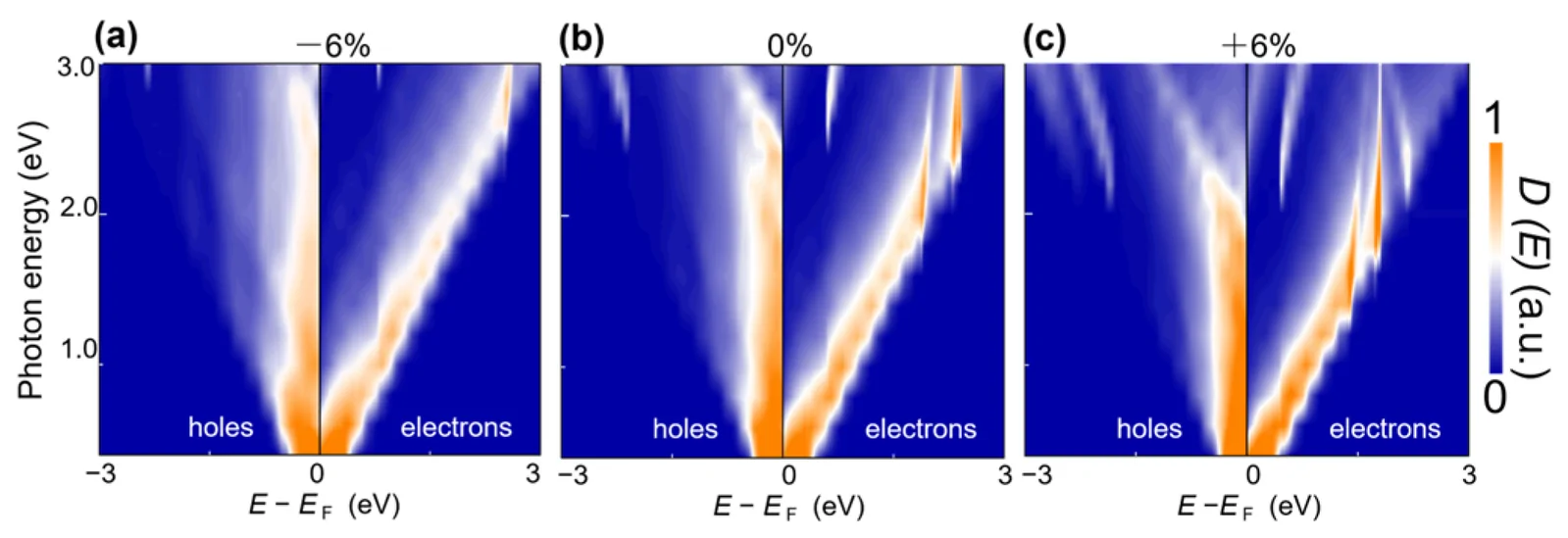
Initial energy distribution of hot carriers [D(*E*)] generated from indirect and direct transitions under (**a**) −6% compressive strain, (**b**) unstrained, and (**c**) +6% tensile strain. The Fermi level (*E*_F_) is set to zero, the hot holes are below *E*_F_, and the hot electrons are above *E*_F_.

We further investigate the hot carrier energy distribution under compressive and tensile strains, which is determined by the energies of the initial (hot holes) and final states (hot electrons). Figure 3 presents the hot carrier energy distribution, encompassing both indirect and direct transitions. Figure 3a reveals that −6% strain reduces the generation of hot carriers in the high-energy region, a phenomenon associated with strain-induced modifications in the electronic structure. For instance, compressive strain enhances band delocalization and decreases the DOS, which collectively reduce hot carrier generation. In contrast, under +6% strain, the generation of hot carriers in the high-energy region is enhanced. However, a higher population of high-energy hot carriers leads to more pronounced thermalization, which in turn increases hot carrier collisions, reduces the MFP, and thereby decreases the hot carrier injection efficiency. Therefore, although compressive strain affects hot carrier generation, it is inferred that compressive strain may enhance the electrical performance of hot carriers.

### 3.3. Hot Carrier Transport and Injection

**Figure 4:**
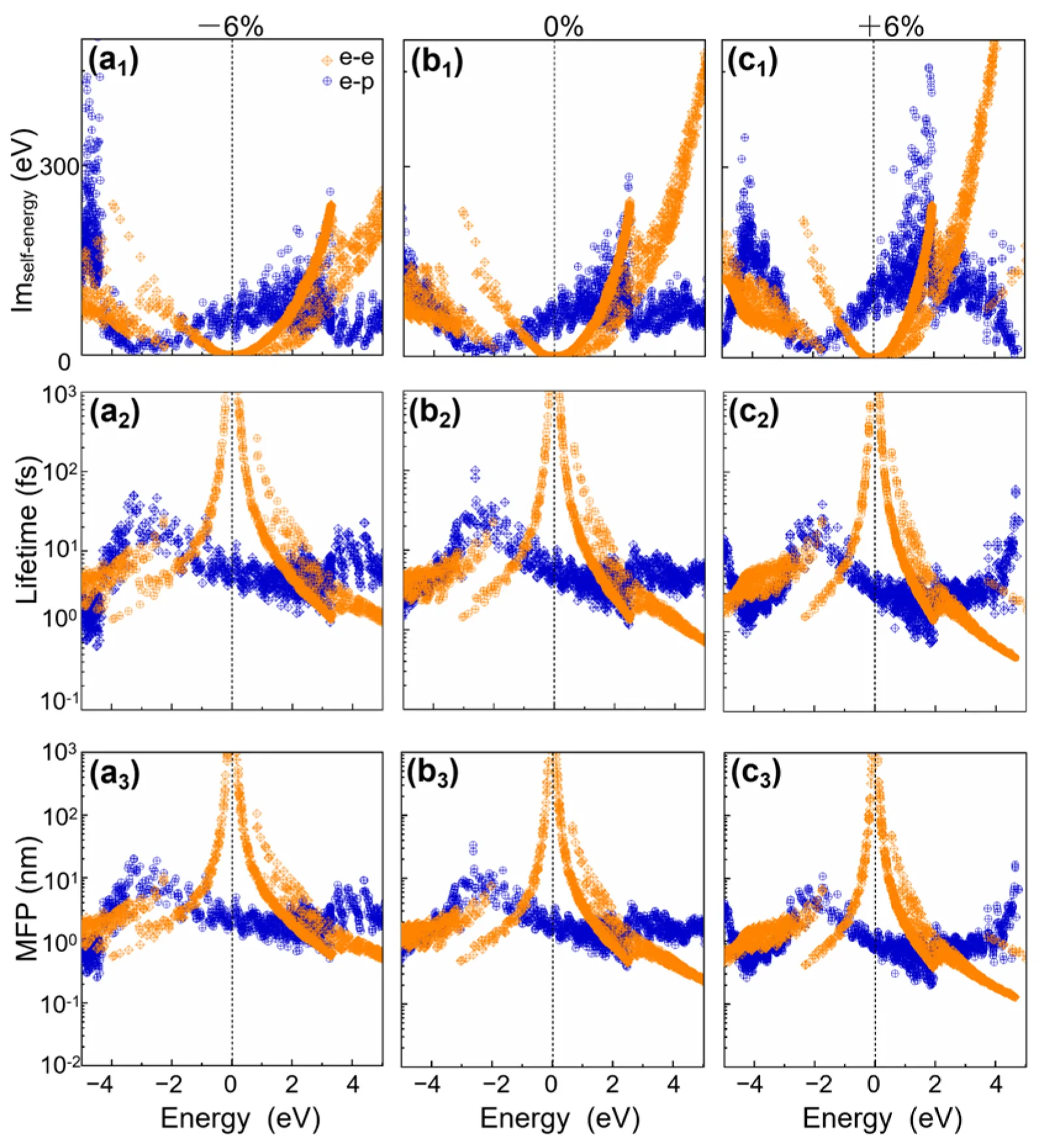
Imaginary part of quasi-particle self-energy (**a_1_**,**b_1_**,**c_1_**), lifetime (**a_2_**,**b_2_**,**c_2_**), and mean free path (**a_3_**,**b_3_**,**c_3_**) of hot carriers from electron–electron and electron–phonon scatterings in TiN under −6%, 0%, and +6% strains. The zero energy in (**a**–**c**) are set as *E*_F_.

Having previously demonstrated that −6% compressive strain reduces the generation of hot carriers in the high-energy region, we now investigate the transport properties of hot carriers under different strains to determine how strain affects the injection efficiency of hot carrier devices. First, Figure 4(a_1_–c_1_) illustrates the variations in the imaginary part of the quasiparticle self-energy (IQS) for S_e-e_ and S_e-p_ under strain. It is observed that under −6% strain, both IQS values of S_e-e_ and S_e-p_ decrease compared to the pristine case, particularly for S_e-e_ in the high-energy region. This is primarily attributed to the reduced DOS under −6% strain, especially above the Fermi level. In contrast, under +6% strain, both IQS of S_e-e_ and S_e-p_ increase relative to the pristine case. Next, we examine the hot carrier lifetime. Figure 4(a_2_–c_2_) shows the lifetimes of carriers subject to S_e-e_ and S_e-p_. Compared to the pristine case, −6% strain leads to an increase in the lifetime under S_e-e_, particularly in the hot electron region. This is mainly due to the significant reduction in the DOS under −6% strain, which in turn reduces S_e-e_. Conversely, the opposite trend is observed under +6% strain. Finally, we consider the MFP of hot carriers (as shown in Figure 4(a_3_–c_3_), which directly impacts the device’s hot carrier injection efficiency. Under −6% strain, the MFP values under S_e-e_ and S_e-p_ increase compared to the pristine case, especially in the hot electron region. In contrast, under +6% strain, the increased DOS causes a decrease in the MFP for S_e-e_ and S_e-p_. Therefore, −6% strain enhances the transport properties of hot electrons in TiN.

**Figure 5:**
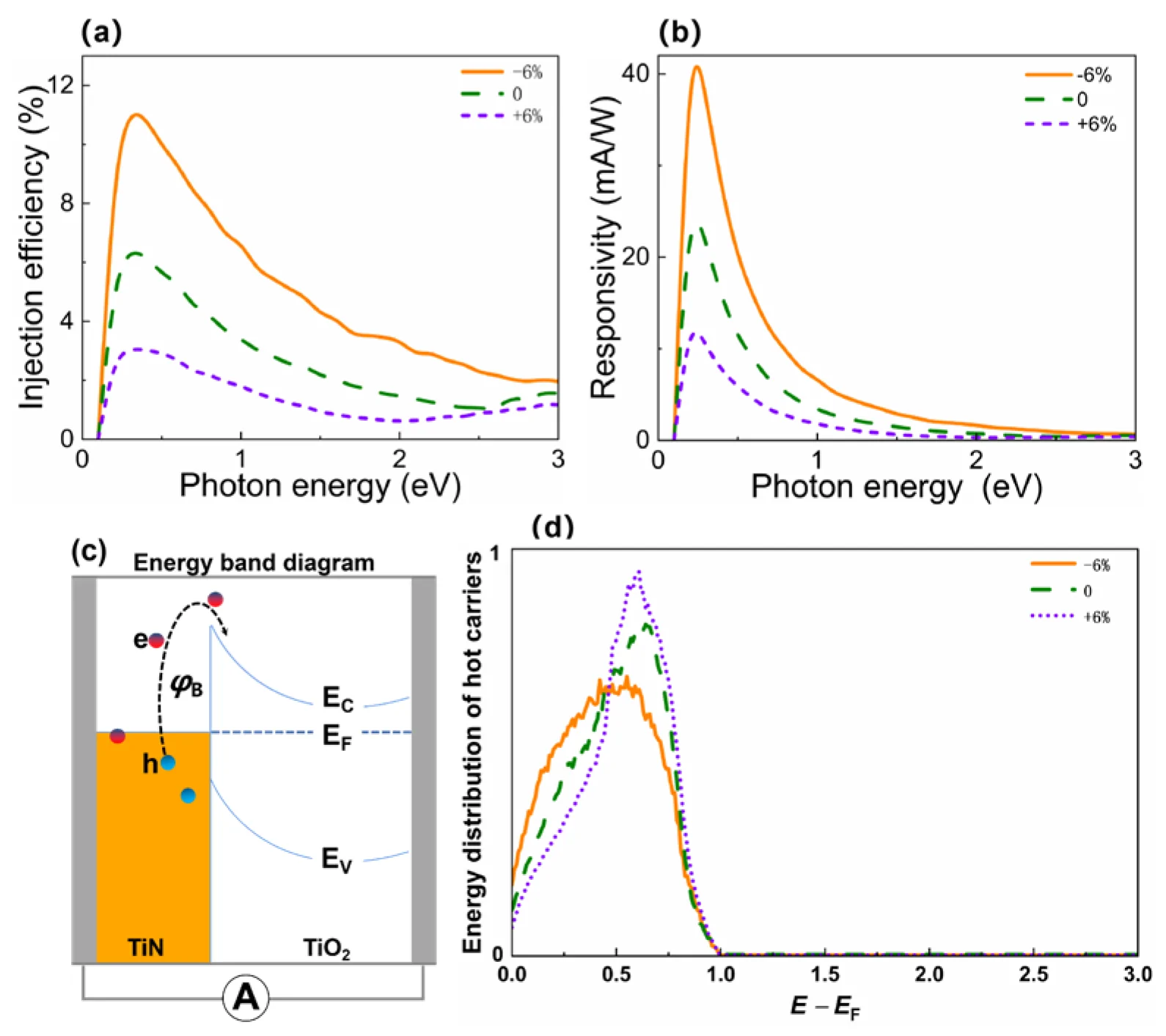
(**a**,**b**) Injection efficiency and responsivity of TiN-TiO_2_ hot carrier photodetector in a planar configuration, where −6%, 0% and +6% strains are comparably studied. (**c**) Energy band diagram. (**d**) Initial energy distribution at *hv* = 0.8 eV (1550 nm).

Finally, we investigate the performance of the TiN/TiO_2_ hot electron photodetector under different strains. A simple planar TiN/TiO_2_ hot electron photodetector is taken as a representative example, with its device schematics and band diagram illustrated in Figure 5a. The Schottky barrier height of the TiN/TiO_2_ junction is 0.1 eV [11], and the TiN layer has a thickness of 10 nm. We employ the Monte Carlo method to simulate the hot carrier injection efficiency and responsivity of the device [38]. The hot electron injection efficiency and responsivity under −6%, +6% strain, and unstrained conditions are presented in Figure 5b,c. We find that −6% strain significantly enhances the injection efficiency and responsivity by a factor of 1.3–2.4 within the photon energy range of 0.1–3 eV. Conversely, +6% strain degrades these two metrics by an analogous factor of 1.3–2.4 over the identical spectral interval. These results indicate that while −6% strain reduces hot electron generation, it simultaneously improves the transport properties of hot electrons, thereby enhancing overall device performance. In contrast, +6% strain deteriorates device performance.

To elucidate the underlying mechanism by which compressive strain enhances device performance, we analyze the evolution of key physical parameters of TiN under varying strains at a photon energy of 0.8 eV (corresponding to the telecommunication wavelength of 1550 nm), as summarized in Table 1. Our results show that both the DOS and hot electron generation rate of TiN decrease under a −6% strain. Notably, as shown in Figure 5d, under −6% strain, the population of hot electrons increases in the energy range near the Schottky barrier (0.1~0.5 eV) but decreases at higher energies (>0.5 eV above the Fermi level). This reduction in the high-energy region suppresses S_e-e_, thereby extending the hot electron lifetime and mean free path, which ultimately enhances injection efficiency. We further benchmark our results against previously reported 1550 nm photodetector performances, as also listed in Table 1. The TiN/ZnO/TiN heterostructure reported by Wang et al. exhibits a responsivity of 0.23 mA/W [39]; the TiN/Ge device achieves 11.5 mA/W [40]; and Au/MoS_2_-based devices reach a responsivity of ~10 mA/W [41]. Our reported responsivity of 9.81 mA/W is comparable to these values. It is important to note that the model does not account for the impacts of interfacial defects, device structural non-idealities, temperature effects, and related practical factors on device performance; nevertheless, it successfully captures the strain-induced trends in hot carrier dynamics.

## 4. Conclusions

In summary, we employ first-principles calculations and Monte Carlo simulations to demonstrate that compressive strain can modulate the electronic structure of TiN, thereby tuning the generation, transport, and injection properties of hot carriers in TiN. Our study reveals that under −6% strain, the DOS of TiN decreases and broadens. This leads to a reduction in hot electron generation in TiN, particularly in the high-energy region above the Schottky barrier, which in turn diminishes electron–electron scattering, thereby enhancing the hot electron lifetime and MFP. Conversely, +6% strain increases and localizes the TiN DOS, resulting in greater hot electron generation in the high-energy regime. This intensifies electron–electron scattering, shortening the hot electron lifetime and MFP. Finally, taking a planar TiN/TiO_2_ hot electron photodetector as a prototype, we investigate the photodetector performance through numerical simulations. The simulation results show that within the photon energy range of 0.1–3 eV, −6% strain enhances the photodetector’s responsivity and injection efficiency by a factor of 1.3–2.4. These predictions, derived from idealized interface models, provide a theoretical foundation for strain-engineered device optimization; the relative trends and underlying physics are reliable, though absolute values may shift with experimental conditions, such as interfacial defects and temperature effects, which are not included here. Nonetheless, this study offers essential theoretical guidance for the strain-engineered design of TiN-based optoelectronic devices.

## Figures and Tables

**Table 1:** The variation in physical parameters, where 6%, 0% and +6% strains are comparably studied in 1550 nm.

The Characteristic Value at 1550 nm	−6%	0	+6%
Density of states (state/eV)	0.82	1.46	2.08
Generation efficiency (a.u.)	0.03	0.06	0.09
Lifetime (fs)	29.41	16.94	12.49
Mean free path (nm)	11.79	5.67	3.44
Injection efficiency (%)	7.85	4.24	2.21
Responsivity(mA/W)	9.81	5.30	2.76
TiN/ZnO/TiN Responsivity (mA/W) [39]	0.23		
TiN/Ge Responsivity (mA/W) [40]	11.5		
Au/MoS_2_(mA/W) [41]	10		

## Data Availability

The data that support the findings of this study are available from the corresponding author upon reasonable request.
